# Type 2 diabetes mellitus, blood cholesterol, triglyceride and colorectal cancer risk in Lynch syndrome

**DOI:** 10.1038/s41416-019-0580-9

**Published:** 2019-09-25

**Authors:** S. Ghazaleh Dashti, Wing Yan Li, Daniel D. Buchanan, Mark Clendenning, Christophe Rosty, Ingrid M. Winship, Finlay A. Macrae, Graham G. Giles, Sheetal Hardikar, Xinwei Hua, Stephen N. Thibodeau, Jane C. Figueiredo, Graham Casey, Robert W. Haile, Steven Gallinger, Loïc Le Marchand, Polly A. Newcomb, John D. Potter, Noralane M. Lindor, John L. Hopper, Mark A. Jenkins, Aung Ko Win

**Affiliations:** 1 Centre for Epidemiology and Biostatistics, Melbourne School of Population and Global Health, The University of Melbourne, Parkville, VIC 3010 Australia; 2 Victorian Comprehensive Cancer Centre, University of Melbourne Centre for Cancer Research, Melbourne, VIC 3000 Australia; 3 Cancer Epidemiology and Intelligence Division, Cancer Council Victoria, Melbourne, VIC 3004 Australia; 4 Genetic Medicine, Royal Melbourne Hospital, Parkville, VIC 3050 Australia; 5 Colorectal Oncogenomics Group, Department of Clinical Pathology, The University of Melbourne, Parkville, VIC 3010 Australia; 6 Envoi Specialist Pathologists, Brisbane, QLD 4059 Australia; 7 Faculty of Medicine, University of Queensland, Brisbane, QLD 4006 Australia; 8 Department of Medicine, The University of Melbourne, Parkville, VIC 3010 Australia; 9 Colorectal Medicine and Genetics, Royal Melbourne Hospital, Parkville, VIC 3050 Australia; 10 Department of Population Health Sciences, University of Utah, Salt Lake City, UT 84112 USA; 11 Population Sciences, Huntsman Cancer Institute, Salt Lake City, UT 84112 USA; 12 Public Health Sciences Division, Fred Hutchinson Cancer Research Center, Seattle, WA 98109 USA; 13 School of Public Health, University of Washington, Seattle, WA 98195 USA; 14 Molecular Genetics Laboratory, Department of Laboratory Medicine and Pathology, Mayo Clinic, Rochester, MN 55455 USA; 15 Samuel Oschin Comprehensive Cancer Institute, Cedars-Sinai Medical Center, Los Angeles, CA 90048 USA; 16 Department of Preventive Medicine, Keck School of Medicine, University of Southern California, Los Angeles, CA 90032 USA; 17 Center for Public Health Genomics, University of Virginia, Charlottesville, VA 22908 USA; 18 Lunenfeld Tanenbaum Research Institute, Mount Sinai Hospital, University of Toronto, Toronto, ON M5G 1×5 Canada; 19 University of Hawaii Cancer Center, Honolulu, Hawaii 96813 USA; 20 Centre for Public Health Research, Massey University, Wellington, 6140 New Zealand; 21 Department of Health Science Research, Mayo Clinic Arizona, Scottsdale, AZ 85259 USA

**Keywords:** Colon cancer, Cancer epidemiology

## Abstract

**Background:**

Type 2 diabetes mellitus and high total cholesterol and triglycerides are known to be associated with increased colorectal cancer risk for the general population. These associations are unknown for people with a germline DNA mismatch repair gene mutation (Lynch syndrome), who are at high risk of colorectal cancer.

**Methods:**

This study included 2023 (56.4% female) carriers with a mismatch repair gene mutation (737 in *MLH1*, 928 in *MSH2*, 230 in *MSH6*, 106 in *PMS2*, 22 in *EPCAM*) recruited by the Colon Cancer Family Registry between 1998 and 2012. Weighted Cox regression was used to estimate the hazard ratios (HR) and 95% confidence intervals (CI) for the associations between self-reported type 2 diabetes, high cholesterol, triglyceride and colorectal cancer risk.

**Results:**

Overall, 802 carriers were diagnosed with colorectal cancer at a median age of 42 years. A higher risk of colorectal cancer was observed in those with self-reported type-2 diabetes (HR 1.92; 95% CI, 1.03–3.58) and high cholesterol (HR 1.76; CI 1.23–2.52) compared with those without these conditions. There was no evidence of high triglyceride being associated with colorectal cancer risk.

**Conclusion:**

For people with Lynch syndrome, self-reported type-2 diabetes mellitus and high cholesterol were associated with increased colorectal cancer risk.

## Introduction

Lynch syndrome is caused by a germline mutation in one of the DNA mismatch repair (MMR) genes *MLH1*, *MSH2*, *MSH6* and *PMS2* or a deletion in *EPCAM*.^1–5^ Lynch syndrome is estimated to be present in one in 279 people in the general population^6^ and to cause ~2–5% of all colorectal cancers.^7,8^ People with Lynch syndrome are at increased risk of various types of cancers, mainly colorectal cancer. The average cumulative risk of colorectal cancer to age 70 years (95% confidence interval (CI)), for male and female mutation carriers, respectively, has been estimated to be 34% (25–50%) and 36% (25–51%) for *MLH1*,^9^ and 47% (36–60%) and 37% (27–50%) for *MSH2*,^9^ 22% (14–32%) and 10% (5–17%) for *MSH6*,^10^ and 7.2% (4.5–13%) and 6.4% (3.8–12%) for *PMS2*.^11^ Further, there is evidence that the risk varies substantially even across carriers of a mutation in the same gene, with the majority of carriers being either at only modestly increased risk or at very high risk, rather than being clustered around the ‘average’ risk.^9^ These findings are consistent with that other genetic or environmental and lifestyle factors may modify cancer risk in people with Lynch syndrome.^9,12^

Previous epidemiological studies have shown that, for the general population, diabetes mellitus, particularly type 2, is associated with an increased risk of colorectal cancer.^13,14^ Similarly, there is evidence that high blood levels of total cholesterol and triglyceride increase the risk of colorectal cancer.^15,16^ Investigating whether such associations exist for people with Lynch syndrome is important for predicting risk for targeted screening and a better understanding of carcinogenesis and could provide opportunities to reduce cancer risk in this high-risk population. In the current study, we report the associations between self-reported diagnosis of diabetes mellitus, high cholesterol and high triglyceride, and the risk of colorectal cancer for people with Lynch syndrome, using a large dataset from the Colon Cancer Family Registry.

## Materials and methods

### Study sample

The study sample included carriers of heterozygous germline pathogenic mutations in MMR genes recruited by the Colon Cancer Family Registry (CCFR) between 1998 and 2012. Detailed description of the study design and recruitment by each site of the CCFR have been previously published^17,18^ and can be found at http://www.coloncfr.org/. Briefly, the CCFR recruitment protocols fall broadly into two main categories: population-based and clinic-based. Through population-based cancer registries in the United States (Hawaii, Minnesota, Puget Sound, California, Arizona, Colorado, New Hampshire, and North Carolina), Australia (Victoria) and Canada (Ontario), individuals with recent diagnosis of colorectal cancer were recruited regardless of family histories or stratified by family histories. Through familial cancer clinics in the United States (Mayo Clinic, Rochester, Minnesota, and Cleveland Clinic, Cleveland, Ohio), Canada (Ontario), Australia (Brisbane, Melbourne, Adelaide, Sydney, and Perth), and New Zealand (Auckland), individuals with or without colorectal cancer were recruited because they were part of families with colorectal cancer. Relatives of probands were recruited according to pre-specified rules of recruiting centres, with prior permission obtained from the probands.^17^ Informed consent was obtained from all study participants and the study protocol was approved by the research ethics review boards of each recruitment site.

### Data collection

At the time of recruitment, information on demographics and personal characteristics such as education, height, current weight and weight at age 20, lifestyle factors such as smoking, alcohol consumption, physical activity, diet, medical history including having received diagnosis of  diabetes mellitus, high cholesterol, and high triglyceride, age at diagnosis of these conditions and any medication used to control them, medication history such as aspirin, calcium supplement intake, cancer screening history, and any previous surgery were obtained from all participating probands and relatives. The questionnaire was standardised across all CCFR sites and was administered via in-person interviews, telephone interviews, or mailed paper questionnaires (questionnaire details can be found at http://coloncfr.org/questionnaires). Personal and family history of all cancers (except non-melanoma skin cancers) and age diagnosis were also collected. When possible, cancer diagnoses were verified using pathology reports, medical records, and/or cancer registries. Blood samples were collected from all consenting participants and tumour tissues from those diagnosed with colorectal cancer.

### MMR gene mutation testing

Screening for germline mutations in *MLH1, MSH2, MSH6, PMS2* and *EPCAM* was performed for all population-based probands who had a colorectal tumour displaying high levels of microsatellite instability (MSI) or a loss of expression of one or more of the MMR proteins by immunohistochemistry (IHC) and, for the youngest-onset colorectal cancer-affected participant from each clinic-based family, regardless of MSI or MMR-protein expression status.^17^ MMR gene mutation testing has been described in detail previously.^6^ Where available, blood samples from the relatives of probands with a pathogenic mutation^6^ were tested for the specific MMR gene mutation identified in the proband (predictive testing).

### Definitions of exposures

The exposures of interest were diabetes mellitus, defined as present if a participant answered “yes” to “has a doctor ever told you that you had diabetes (excluding diabetes which you had only during pregnancy)?”; high cholesterol, defined as present if a participant answered “yes” to “has a doctor ever told you that you had high cholesterol?”; and high triglycerides defined as present if a participant answered “yes” to “has a doctor ever told you that you have high levels of triglycerides in your blood?”. Ages at diagnoses of these conditions were self-reported. The three exposure variables were treated as time-varying exposures, e.g. carriers were categorised as not having diabetes prior to age of diagnosis of diabetes mellitus and as having diabetes mellitus at age of diagnosis of diabetes mellitus and thereafter. Exposure variables were coded as missing if reported age at diagnosis of the condition was missing (two participants with diabetes mellitus, 28 with high cholesterol and 15 with high triglyceride) and as zero if age at diagnosis was after the age at colorectal cancer diagnosis or age at censoring (see below). Information on type of diabetes mellitus was not collected. However, we attempted to identify carriers with type 2 diabetes mellitus by defining them as those diagnosed with diabetes mellitus at age 35 or older, or diagnosed with diabetes mellitus under age 35 and on oral medication.^19^

### Statistical analysis

We included probands and relatives with confirmed pathogenic MMR gene mutations—from both clinic-based and population-based CCFR sites—who had completed the interview at recruitment. For this study, all analyses were restricted to data for exposures, outcome, and covariates collected at the time of recruitment, i.e. at age at interview.

We conducted weighted Cox proportional hazards regression analysis, with age as the time scale, to estimate the association between diabetes mellitus, high cholesterol, and high triglyceride with the risk of colorectal cancer. Time at risk started at age 20 and ended at age at first diagnosis of colorectal cancer, age at polypectomy, diagnosis of non-skin, non-colorectal cancers (excluding non-melanoma skin cancers), or age at interview, whichever occurred first. Diabetes mellitus, high cholesterol, and high triglycerides were fitted as time-varying exposures in the models. Data on self-reported age at diagnosis of the three primary exposures variables was used to define time-varying exposure variables; therefore, exposure time started at age at diagnosis of each condition.

Because some participants were recruited from families with colorectal cancer histories and case probands were tested preferentially for MMR gene mutations, participants included in the analysis were not randomly ascertained in relation to disease status. To minimise bias caused by this non-random ascertainment, we used a “weighted cohort approach” described by Antoniou et al.,^20^ which has been used in similar studies of modifiers for Lynch syndrome.^21–26^ Briefly, we first calculated the incidence rates of colorectal cancer for the MMR mutation carriers for age groups <30, 30–34, 35–39, 40–44, 45–49, 50–54, 55–59, 60–64 and >64 based on colorectal cancer incidence in the general population, averaged for men and women,^27^ and the hazard ratio of colorectal cancer for each MMR gene mutated.^9,10,28^ These incidence rates was then used together with the number and total person-years of observation for affected and unaffected carriers in each age group to calculate the age-specific sampling fractions. Finally, probability weights were calculated based on the sampling fractions and assigned to the affected and unaffected carriers, so that the age-specific proportion of affected carriers in the “synthetic” cohort equalled the proportion of affected carriers in the general population.^29^ The Huber-White robust variance estimation by clustering on family membership was applied to account for possible correlation in risk between carriers from the same family.^30^

Sex, ascertainment method, country of recruitment, education, body mass index (BMI) at age 20, average daily ethanol intake from alcoholic beverages, smoking status, and physical activity were identified as potential confounders. When applicable, potential confounders were also fitted as time-varying covariates. Diabetes mellitus was also included as a confounder in multivariable models treating high cholesterol or high triglyceride as the exposure. We did not condition on high cholesterol or high triglyceride in the models for diabetes mellitus, because these might have been on the causal pathway from diabetes mellitus to colorectal cancer incidence.^31^ Fruit, vegetable and meat intake were not included in the primary analysis, because carriers who were censored or received colorectal cancer diagnosis more than 2 years before interview were missing information for those variables. To test for interactions, the difference in the log-likelihood ratio was assessed after adding a cross-product term between each exposure variable and the potential effect modifiers identified a priori (carrier’s sex and mutated MMR gene).

The number of missing values for all variables are reported in Tables 1 and 2. All analyses were complete case analyses. We did not test for the proportional hazards assumption, because it cannot be interpreted with time-varying exposure.

**Table 1:** Characteristics of people with Lynch syndrome included in the study

	No colorectal cancer (*N* = 1221)	Colorectal cancer (*N* = 802)	Overall (*N* = 2023)
Sex, *n* (%)			
Male	445 (36.4)	437 (54.5)	882 (43.6)
Female	776 (63.6)	365 (45.5)	1141 (56.4)
Study Centres, *n* (%)			
Canada	168 (13.8)	131 (16.3)	299 (14.8)
Australia or New Zealand	712 (58.3)	377 (47.0)	1089 (53.8)
USA	341 (27.9)	294 (36.7)	635 (31.4)
Ascertainment method, *n* (%)			
Clinic	968 (79.3)	524 (65.3)	1492 (73.8)
Population	253 (20.7)	278 (34.7)	531 (26.2)
Proband, *n*(%)			
No	1,001 (82.0)	328 (40.9)	1329 (65.7)
Yes	220 (18.0)	474 (59.1)	694 (34.3)
Age at cancer diagnosis or censoring age (year)			
Median (interquartile range)	41 (32–50)	42 (35–49)	42(33–50)
[range]	[20–85]	[20–76]	[20–85]
Education level, *n* (%)			
Some high school or less	242 (19.8)	189 (23.6)	431 (21.3)
Completed high school/some tertiary study	404 (33.1)	268 (33.4)	672 (33.2)
Vocational/technical school	223 (18.3)	126 (15.7)	349 (17.3)
University degree	338 (27.7)	205 (25.6)	543 (26.8)
Missing	14 (1.1)	14 (1.7)	28 (1.4)
Mismatch repair gene mutated, *n* (%)			
* MLH1*	389 (31.9)	348 (43.4)	737 (36.4)
* MSH2*	600 (49.1)	328 (40.9)	928 (45.9)
* MSH6*	161 (13.2)	69 (8.6)	230 (11.4)
* PMS2*	58 (4.8)	48 (6.0)	106 (5.2)
* EPCAM*	13 (1.1)	9 (1.1)	22 (1.1)
Body mass index at age 20, *n* (%)			
<18.5 kg/m^2^	114 (9.3)	62 (7.7)	176 (8.7)
18.5–25.0 kg/m^2^	815 (66.7)	509 (63.5)	1324 (65.4)
25.0–29.9 kg/m^2^	194 (15.9)	152 (19.0)	346 (17.1)
≥30 kg/m^2^	49 (4.0)	43 (5.4)	92 (4.5)
Missing	49 (4.0)	36 (4.5)	85 (4.2)
Regular physical activity,^a^ *n* (%)			
No	53 (4.3)	49 (6.1)	102 (5.0)
Yes	1,128 (92.4)	713 (88.9)	1841 (91.0)
Missing	40 (3.3)	40 (5.0)	80 (4.0)
Cigarette smoking,^b^ *n* (%)			
Never smoker	667 (54.6)	359 (44.8)	1026 (50.7)
Former smoker	274 (22.4)	170 (21.2)	444 (21.9)
Current smoker	275 (22.5)	271 (33.8)	546 (27.0)
Missing	5 (0.4)	2 (0.2)	7 (0.3)
Average daily ethanol intake (grams/day),^c^ *n* (%)			
0	321 (26.3)	200 (24.9)	521 (25.8)
>0–≤14	580 (47.5)	314 (39.2)	894 (44.2)
>14–≤28	113 (9.3)	85 (10.6)	198 (9.8)
>28	89 (7.3)	111 (13.8)	200 (9.9)
Missing	118 (9.7)	92 (11.5)	210 (10.4)
Fruit and vegetable intake (servings/day) 2 years before diagnosed/censored age,^d, f^ *n* (%)			
<2	200 (16.4)	123 (15.3)	323 (16.0)
2.01–3	141 (11.5)	54 (6.7)	195 (9.6)
3.01–4	138 (11.3)	53 (6.6)	191 (9.4)
≥4.01	265 (21.7)	69 (8.6)	334 (16.5)
Missing	477 (39.1)	503 (62.7)	980 (48.4)
Red meat intake (servings/day 2 years before diagnosed/censored age),^e, f^ *n* (%)			
<0.30	222 (18.2)	99 (12.3)	321 (15.9)
0.31–0.60	261 (21.4)	94 (11.7)	355 (17.5)
0.61–0.90	105 (8.6)	48 (6.0)	153 (7.6)
≥0.91	157 (12.9)	66 (8.2)	223 (11.0)
Missing	476 (39.0)	495 (61.7)	971 (48.0)

^a^Regular physical activity defined as any physical activity for at least 30 min per week for at least 3 months

^b^Former smokers defined as carriers who had smoked at least one cigarette per day for at least 3 months and had quit more than 2 years before age at colorectal cancer or censored age; current smokers defined as carriers who had smoked at least one cigarette per day for at least 3 months and continued within 2 years of age at colorectal cancer or censored

^c^Average daily ethanol intake was calculated as weighted average of daily ethanol intake from any alcoholic beverage when carriers were in their 20, 30, 40 and 50 s and above

^d^A serving of fruit defined as 1 medium fresh fruit, or ½ cup of chopped, cooked, or canned fruit, or¼ cup of dried fruit, or 6 ounce of fruit juice; a serving of vegetable defined as 1 cup raw leafy vegetables, or ½ cup of other vegetables, or cooked or chopped raw, 6 ounces of vegetable juice

^e^Aserving of red meat defined as 2–3 ounces of red meat, or a piece of meat about the size of a deck of cards

^f^Carriers who were diagnosed with cancer or censored more than 2 years before interview had missing for this variable

**Table 2:** Diabetes mellitus, high cholesterol, and high triglyceride levels in people with Lynch syndrome included in the study

	No colorectal cancer (*N* = 1221)	Colorectal cancer (*N* = 802)	Overall (*N* = 2023)
Diabetes mellitus, *n* (%)			
No	1182 (96.8)	761 (94.9)	1943 (96.0)
Yes, either type 1 or 2	33 (2.7)	33 (4.1)	66 (3.3)
Yes, type 2^a^	30 (2.5)	30 (3.7)	60 (3.0)
Missing	6 (0.5)	8 (1.0)	14 (0.7)
Medication to control diabetes, *n* (% of diabetics)			
No	7 (21.2)	6 (18.2)	13 (19.7)
Pills	14 (42.4)	10 (30.3)	24 (36.4)
Insulin injection only	6 (18.2)	6 (18.2)	12 (18.2)
Pills and insulin injections	6 (18.2)	11 (33.3)	17 (25.8)
Age at diagnosis of diabetes mellitus (year)			
Median (interquartile range)	51 (44–61)	50 (40–58)	50 (42–60)
Years from diabetes diagnosis to colorectal cancer diagnosis or censoring			
Median (interquartile range)	5 (1–11)	6 (3–10)	5.5 (1–10)
High cholesterol, *n* (%)			
No	1040 (85.2)	697 (86.9)	1737 (85.9)
Yes	160 (13.1)	90 (11.2)	250 (12.4)
Missing	21 (1.7)	15 (1.9)	36 (1.8)
Medication to control high cholesterol, *n* (% of with high cholesterol)			
No	80 (50.0)	47 (52.2)	127 (50.8)
Yes	79 (49.4)	43 (47.8)	122 (48.8)
Missing	1 (0.6)	0	1 (0.4)
Age at diagnosis of high cholesterol (year)			
Median (interquartile range)	50 (41–58.5)	50 (40–58)	50 (41–58)
Years from high cholesterol diagnosis to colorectal cancer diagnosis or censoring			
Median (interquartile range)	4 (1–9)	4 (1–9)	4 (1–9)
High triglyceride, *n* (%)			
No	1111 (91.0)	729 (90.9)	1840 (91.0)
Yes	49 (4.0)	32 (4.0)	81 (4.0)
Missing	61 (5.0)	41 (5.1)	102 (5.0)
Medication to control high triglyceride, *n* (% of with high triglyceride)			
No	35 (71.4)	23 (71.9)	58 (71.6)
Yes	14 (28.6)	7 (21.9)	21 (25.9)
Missing	0	2 (6.3)	2 (6.3)
Age at diagnosis of high triglyceride (year)			
Median (interquartile range)	50 (41–57)	45 (35–53)	48 (40–55)
Years from high triglyceride diagnosis to colorectal cancer diagnosis or censoring			
Median (interquartile range)	49 (1–8)	4 (1–9)	4 (1–9)

^a^Type 2 diabetes defined as diagnosed ≥35 years age or diagnosis <35 years and not on insulin injection only medication

We also undertook additional analyses restricted to carriers who had a colorectal cancer diagnosis or were censored within 5 years before interview; analyses additionally adjusted for consumptions of fruit, vegetable and red meat intake 2 years before colorectal cancer diagnosis or censoring; and analyses without applying the probability weights.

All *p*-values reported are two-sided. Stata version 14.2 was used for statistical analysis.^32^

## Results

A total of 2164 MMR gene mutation carriers were identified across the CCFR. Of these, 65 who had not completed the interview at baseline recruitment, six with missing age at cancer diagnosis, 13 who did not have data on diabetes mellitus, high cholesterol and high triglyceride and 57 who were censored before age 20 were excluded. The final sample comprised 2023 carriers; of whom 694 (34.3%) were probands; 1141 (56.4%) were females, 1492 (73.8%) were recruited from familial cancer clinics; 737 carried a mutation in *MLH1*, 928 in *MSH2*, 230 in *MSH6*, 106 in *PMS2*, and 22 in *EPCAM*. Participants were from 773 families, of which 431 had one affected relative, 148 had more than one affected relative, and 194 had no affected relative. The characteristics of people with Lynch syndrome included in the study are summarised in Table 1. For 802 of the included people, time at risk ended at colorectal cancer diagnosis, for 344 at first polypectomy, for 267 at non-skin non-colorectal cancers diagnosis, and for 610 at interview. The median ages (interquartile range) at colorectal cancer diagnosis and censoring were 42 (35–49) and 41 (32–50) years, respectively. Incidence rate of colorectal cancer from birth was 943 (95% confidence interval (CI) 880–1011) per 100,000 person-years.

Overall, 66 (3.3%) carriers reported having been diagnosed with diabetes mellitus before colorectal cancer diagnoses or censoring (median (interquartile) age at diagnosis 50 (42–60) years). Of these, 60 (91%) were categorised as having type 2 mellitus (the remaining 9 % were not included in analyses that compared colorectal cancer risk in carriers with versus without type 2 diabetes). A total of 250 (12.4%) carriers reported having been diagnosed with high blood cholesterol at a median (interquartile) age of 50 (41–58) years, and 81 (4.0%) reported having been diagnosed with high triglyceride at a median (interquartile) age of 48 (40–55) years (Table 2).

After adjusting for potential confounders, carriers with type 2 diabetes mellitus had a higher risk of colorectal cancer compared with those without diabetes mellitus (HR 1.92; 95% CI, 1.03–3.58). There was also evidence of a positive association between high blood cholesterol and the risk of colorectal cancer (HR 1.76; 95% CI 1.23–2.52). We did not observe evidence for an association between blood triglyceride and colorectal cancer risk for people with Lynch syndrome in the adjusted model (HR 1.42; 95% CI 0.77–2.64) (Table 3). Results were similar when analyses were limited to carriers who reported not taking any medication to control their high cholesterol or high triglyceride (Table 3). The directions and strength of associations were similar when analyses were restricted to carriers censored within 5 years before baseline interview (Supplementary Table 1). There was no statistical evidence for an interaction between these exposure variables and sex or mutated MMR genes (Supplementary Table 2).

**Table 3:** Hazard ratios for association between diabetes mellitus, high cholesterol, high triglyceride and colorectal cancer risk in people with Lynch syndrome included in the study

	No. Cases	Person-years	Univariable analysis		Multivariable analysis	
			HR (95% CI)	*P* value	HR (95% CI)	*P* value
Diabetes mellitus						
No	761	43653	1 (Ref)		1 (Ref)	
Yes, type 1 and type 2	33	560	2.27 (1.41–3.64)	0.001	1.71 (0.98–2.97)	0.06
Yes, classified as type 2^a^	30	447	2.42 (1.46–4.03)	0.001	1.92 (1.03–3.58)	0.04
High cholesterol^b^						
No	697	41844	1 (Ref)		1 (Ref)	
Yes	90	1849	1.51 (1.09–2.10)	0.01	1.76 (1.23–2.52)	0.03
Yes, and not taking any medication to control high cholesterol	47	670	2.16 (1.45–3.21)	<0.001	2.59 (1.65–4.05)	<0.001
High triglyceride^b^						
No	729	41396	1 (Ref)		1 (Ref)	
Yes	32	589	1.72 (0.99–2.98)	0.05	1.42 (0.77–2.64)	0.26
Yes, and not taking any medication to control high triglyceride	25	401	1.99 (1.05–3.77)	0.03	1.86 (0.89–3.91)	0.10

All analysis were complete case analysis. All multivariable models were adjusted for sex, ascertainment, country of recruitment, education, body mass index at age 20, average daily ethanol intake from alcoholic beverages (time-varying), and smoking status (time-varying)

*HR* hazard ratio, *CI* confidence interval

^a^Type 2 diabetes mellitus defined as diabetes mellitus diagnosed ≥35 years age or diagnosis <35 years in carriers reporting not on insulin injection only medication

^b^Models were additionally adjusted for diabetes mellitus status (no, yes type 2, yes other; time-varying)

Analyses that were additionally adjusted for fruit, vegetable and red meat intake 2 years before colorectal cancer diagnosis or censoring yielded results comparable with those from the primary analysis (detailed results not shown). Results from unweighted analyses were similar to those from weighted analyses, although the standard errors were smaller, and the 95% CIs were narrower in the former (detailed results not shown).

## Discussion

In this study of people with Lynch syndrome, we observed a higher risk of colorectal cancer associated with self-reported diagnosis of type 2 diabetes mellitus and high blood cholesterol compared with those without these conditions. There was no evidence that these associations differed by sex or the mutated MMR gene.

A meta-analysis of 30 prospective studies reported that diabetes mellitus (most studies did not distinguish the type) increased the risk of colorectal cancer in the general population (summary RR 1.27; 95% CI 1.21–1.34).^14^ Our estimated 95% confidence interval overlapped with what was estimated by the meta-analysis, suggesting that the increased risk of colorectal cancer for people with Lynch syndrome is likely to be comparable with that for the general population. In this study, we had a limited number of people with type 2 diabetes mellitus (66 overall (3.3%) and 33 with colorectal cancer), which might explain our wide confidence interval. Low prevalence of diabetes in our relatively young cohort (median age at colorectal cancer diagnosis or censoring was 42 years (interquartile range 33–50)) was comparable with the under 5% prevalence reported in people younger than 45-years-old in the USA,^33^ Canada^34^ and Australia.^35^

For the general population, several underlying mechanisms have been proposed to explain the role of diabetes mellitus on cancer risk.^36–38^ A characteristic of type 2 diabetes mellitus is insulin resistance and hyperinsulinemia. Insulin may increase the risk of cancer through direct and indirect (via increased free insulin-like growth factor-1) mitogenic and anti-apoptotic properties.^36^ Hyperglycaemia might also increase cancer risk as glucose provides fuel to cancer cells and increases oxidative stress and inflammatory cytokines.^39^ The colorectal carcinogenesis is complex, heterogenous and associated with multiple factors including genetic, epigenetic and environmental factors. Most of Lynch syndrome-related colorectal cancers developed through the MSI pathway which may affect genes that control cell growth (transforming growth factor [TGF] beta and insulin-like growth factor [sIGF] receptors), regulate apoptotic cell death (Caspase 5, Bax), and some of the DNA MMR genes themselves (*MSH3*, *MSH6*). Accumulation of mutations in these cancer-related genes is assumed to drive the carcinogenesis in Lynch syndrome. Evidence suggesting that the effect of type 2 diabetes mellitus would be different on tumours with and without MSI-high is currently lacking. However, a study of 1116 stage-II colon cancer cases observed diabetes mellitus in a comparable proportion of cases with and without MSI-high tumours.^40^

A mendelian randomisation study has shown an increased risk of colorectal cancer with genetically determined higher levels of total cholesterol (odds ratio (OR) per unit increase in genetic risk score 1.46; 95% CI, 1.20–1.79) for the general population.^16^ Previous epidemiological studies have also reported a higher risk of colorectal cancer in association with high versus low levels of total cholesterol (summary RR from a pooled analysis of 10 prospective studies 1.11; 95% CI 1.01–1.21).^15^ Our estimated 95% confidence interval for people with Lynch syndrome from this study overlapped with that with that of the pooled estimate. The mechanisms linking high cholesterol levels to colorectal cancer risk remain speculative; it is suggested that cholesterol may exert carcinogenic effects by increasing oxidative stress and inflammation and compromising immunosurveillance.^16^

High blood triglyceride has been reported to be associated with an increased risk of colorectal cancer for the general population (summary RR from a pooled analysis of 9 prospective studies 1.18; 95% CI 1.04–1.34).^15^ In our study, there was no evidence of an association between high triglyceride and colorectal cancer risk (HR 1.07; 95% CI 0.55–2.09). However, the absence of evidence in our study might have been due to lack of sufficient statistical power to identify any association.

To the best of our knowledge, this is the first study to date assessing the associations between diabetes mellitus, high blood cholesterol and triglyceride and colorectal cancer risk for people with Lynch syndrome. Data for this study were collected using standardised questionnaires and protocols and comprehensive high-quality genetic testing was undertaken to identify MMR mutation carriers in the CCFR.^17^ Self-reported age at diagnosis of the three primary exposures allowed us to analyse these variables as time-varying and thus minimise immortal time bias.^41^ The application of a weighted cohort approach reduced the ascertainment bias of mutation carriers on the basis of their disease status or being part of colorectal cancer families.^20^

A limitation of our study was possible error in measurements of exposures (and other confounding variables), because these were based on self-reported questionnaires. A study of 2037 residents in Minnesota has provided evidence on a substantial agreement (over 95%) between self-reported diabetes mellitus status and medical records.^42^ This agreement may be lower for high cholesterol and triglycerides.^43^ Because the current study included data from the baseline interview at which study participants were aware of their cancer status, this measurement error might have been differential with respect to outcome (recall bias), biasing the results towards or away from the null. Since information on the type of diabetes mellitus was not collected in the CCFR, we classified the type using the self-reported age at diagnosis and type of medication used for diabetes control, based on suggested criteria in a systematic review on clinical characteristic for differentiating type 1 and type 2 diabetes.^19^ Using this approach, 91% of our study participants with diabetes mellitus were classified as having type 2 diabetes, which is consistent with the prevalence in the population.^44^

We explored the potential impact of survival bias on our results by performing additional analyses restricted to carriers who had a cancer diagnosed or were censored within 5 years before baseline interview. Findings from these analyses were not different from the primary analyses. Another limitation of this study is the possibility of residual confounding by factors that we were not able to adjust for in the analyses. We were not able to include fruit, vegetable, and red meat intake in our primary analysis, because carriers who were diagnosed with colorectal cancer or censored more than 2 years before interview had missing values for these variables (the questionnaire asked about these factors 2 years before interview). When these variables were adjusted for in multivariable models as sensitivity analyses, the estimates did not substantially change in direction and strength, compared with those from primary analysis. Although we did not have sufficient statistical power to investigate the association between the exposure variables and colorectal cancer risk by the mutated MMR gene, our test for interaction provided no evidence of effect modification by the mutated gene.

In summary, our study provides evidence that self-reported type 2 diabetes and high cholesterol are associated with an increased risk of colorectal cancer in Lynch syndrome. These associations do not appear to be different across the mutated MMR genes or between male and female carriers. Our finding is consistent with that these are additional risk factors that could be fruitfully monitored and controlled in people with Lynch syndrome, who are at high risk of colorectal cancer.

## Supplementary information

Supplementary Tables

## Data Availability

The data supporting the results reported in this article are available from the Colon Cancer Family Registry (https://www.coloncfr.org/). The data are not publicly available and are subject to data use agreement.
