# Supplementary material for: Type 2 diabetes mellitus, blood cholesterol, triglyceride and colorectal cancer risk in Lynch syndrome
Source: Br J Cancer. 2019 Sep 25;121(10):869–76. doi: 10.1038/s41416-019-0580-9 (PMC6888855; doi:10.1038/s41416-019-0580-9)
Supplement: Supplementary file 1 — Supplementary Tables [file 41416_2019_580_MOESM1_ESM.docx]

| **Supplementary Table 1. Hazard ratios for association between diabetes mellitus, high cholesterol, high triglyceride and colorectal cancer and colorectal cancer risk in people with Lynch syndrome included in the study; analyses limited to participants diagnoses with colorectal cancer or censored within 5 years before baseline interview** | | | | |
| --- | --- | --- | --- | --- |
|  | **No. Cases** | **Person-years** | **Multivariable analysis** |  |
|  |  |  | **HR (95% CI)** | **P value** |
| **Diabetes mellitus** | | | | |
| No | 387 | 27934 | 1 (Ref) |  |
| Yes, type 1 & type 2 | 24 | 466 | 1.81 (1.01 – 3.22) | 0.05 |
| Yes, type 2 ^a^ | 21 | 353 | 2.01 (1.05 – 3.89) | 0.04 |
| **Hyper cholesterol ^b^** | | | | |
| No | 348 | 26767 | 1 (Ref) |  |
| Yes | 61 | 1384 | 2.10 (1.42 – 3.10) | <0.001 |
| Yes, and not taking any medication to control high cholesterol | 32 | 564 |  |  |
| **High triglyceride ^b^** | | | | |
| No | 381 | 26867 | 1 (Ref) |  |
| Yes | 18 | 411 | 1.33 (0.64 – 2.76) | 0.44 |
| Yes, and not taking any medication to control high triglyceride | 25 | 294 |  |  |
| Abbreviations: HR, hazard ratio; CI confidence interval. All analysis were complete case analysis. All multivariable models were adjusted for sex, ascertainment, country of recruitment, education, body mass index at age 20, average daily ethanol intake from alcoholic beverages (time-varying), and smoking status (time-varying). ^a^ Type 2 diabetes mellitus defined as diabetes mellitus diagnosed ≥35 years age or diagnosis<35 years in carriers reporting not on insulin injection only medication. ^b^ Models were additionally adjusted for diabetes mellitus status (no, yes type 2, yes other; time-varying). | | | | |

| **Supplementary Table 2. Assessment of interaction between exposure variables and potential effect modifiers** | | |
| --- | --- | --- |
|  | ***P* Value from likelihood ratio test** | |
|  | **Sex** | **Mutated MMR gene** |
| **Diabetes mellitus** | 0.94 | 0.99 |
| **High cholesterol** | 0.58 | 0.43 |
| **High triglyceride** | 0.45 | 0.35 |
